# Adenovirus Delivered Short Hairpin RNA Targeting a Conserved Site in the 5′ Non-Translated Region Inhibits All Four Serotypes of Dengue Viruses

**DOI:** 10.1371/journal.pntd.0001735

**Published:** 2012-07-24

**Authors:** Anil Babu Korrapati, Gokul Swaminathan, Aarti Singh, Navin Khanna, Sathyamangalam Swaminathan

**Affiliations:** Recombinant Gene Products Group, International Centre for Genetic Engineering and Biotechnology, New Delhi, India; Colorado State University, United States of America

## Abstract

**Background:**

Dengue is a mosquito-borne viral disease caused by four closely related serotypes of Dengue viruses (DENVs). This disease whose symptoms range from mild fever to potentially fatal haemorrhagic fever and hypovolemic shock, threatens nearly half the global population. There is neither a preventive vaccine nor an effective antiviral therapy against dengue disease. The difference between severe and mild disease appears to be dependent on the viral load. Early diagnosis may enable timely therapeutic intervention to blunt disease severity by reducing the viral load. Harnessing the therapeutic potential of RNA interference (RNAi) to attenuate DENV replication may offer one approach to dengue therapy.

**Methodology/Principal Findings:**

We screened the non-translated regions (NTRs) of the RNA genomes of representative members of the four DENV serotypes for putative siRNA targets mapping to known transcription/translation regulatory elements. We identified a target site in the 5′ NTR that maps to the 5′ upstream AUG region, a highly conserved *cis*-acting element essential for viral replication. We used a replication-defective human adenovirus type 5 (AdV5) vector to deliver a short-hairpin RNA (shRNA) targeting this site into cells. We show that this shRNA matures to the cognate siRNA and is able to inhibit effectively antigen secretion, viral RNA replication and infectious virus production by all four DENV serotypes.

**Conclusion/Significance:**

The data demonstrate the feasibility of using AdV5-mediated delivery of shRNAs targeting conserved sites in the viral genome to achieve inhibition of all four DENV serotypes. This paves the way towards exploration of RNAi as a possible therapeutic strategy to curtail DENV infection.

## Author Summary

Dengue is a mosquito-borne viral disease that threatens nearly half the global population. The symptoms of this disease, caused by four closely related Dengue viruses, range from mild fever to potentially fatal haemorrhagic fever and shock. There is neither a preventive vaccine nor an effective antiviral therapy against the disease. The difference between severe and mild disease appears to be dependent on the viral load. Reducing the virus levels in the bloodstream through therapeutic intervention may be associated with favourable prognosis. We investigated the feasibility of destroying dengue virus genomic RNA using a phenomenon known as RNA interference, in which the RNA-cleaving activity of a cellular enzyme complex is directed to a site in the target RNA, using a short complementary RNA known as small interfering RNA. We used adenovirus, a common cold virus, to deliver a small interfering RNA complementary to a conserved region just adjacent to the initiator codon in the dengue virus RNA. We found that this could inhibit viral RNA multiplication, expression of viral proteins and the secretion of infectious virus. Importantly, our results showed that the adenovirus delivered small interfering RNA which could inhibit all four types of dengue viruses.

## Introduction

The genus *Flavivirus*, of the family *Flaviviridae*, includes several vector-borne viruses of which the four serotypes of dengue viruses (DENV-1, -2, -3 and -4) have emerged as the most significant of current challenges to global public health [1], [2]. The DENVs have single-stranded ∼11 kilobase (kb) long 5′capped RNA genomes of positive polarity, containing a single open reading frame (ORF), flanked by ∼100 nucleotide (nt) 5′ and ∼450 nt 3′ non-translated regions (NTRs) [3]. Upon infection of susceptible cells by DENV, the ORF is translated into a single ∼3400 amino acid (aa) residue polyprotein precursor which matures into 3 structural proteins, the capsid, envelope and membrane, and 7 non-structural (NS) proteins, NS1, 2a, 2b, 3, 4a, 4b and 5. This is followed by replication, mediated principally by NS3 and NS5, of the sense genomic RNA through a complementary antisense RNA intermediate [3]. Infection with any of the four DENVs can cause mild and often self-limiting dengue fever (DF) or severe, potentially fatal dengue haemorrhagic fever (DHF) and dengue shock syndrome (DSS) [4]. The severe manifestations of dengue disease, which are more common in hyper-endemic areas during secondary infection with a heterologous DENV serotype, have been correlated to high levels of viremia [5], [6]. Dengue disease which is spread to humans by mosquitoes, threatens almost half the global population. The World Health Organization estimates indicate that globally there are ∼50 million DENV infections each year, with ∼500,000 of these resulting in DHF/DSS, claiming ∼12,500 lives [4]. Though promising live attenuated dengue vaccine candidates are currently in clinical trials, the complex role of the immune system in dengue pathogenesis and viral interference among the four serotypes continue to pose challenging hurdles [1], [2]. Further, no antiviral therapy is currently available to treat DENV infections.

From the perspective of antiviral therapy, the discovery of gene silencing by RNA interference (RNAi) has provided a novel tool. RNAi, first identified to serve an antiviral defence role in plants and insects [7] is an evolutionarily conserved phenomenon that utilizes multiple mechanisms to control eukaryotic gene expression and function through sequence complementarity. One of the effectors of RNAi is small interfering RNA (siRNA), which is taken up by a cytoplasmic RNA-induced silencing complex (RISC). The siRNA programs the endonuclease activity of RISC to cleave target mRNAs that share sequence identity with it, thereby resulting in specific silencing of gene expression [8], [9]. The rapid emergence of RNAi as a potential therapeutic strategy has been catalysed by the demonstration that it could be induced in mammalian cells with synthetic siRNA [10] or with short hairpin RNA (shRNA)-encoding plasmid [11], [12] and viral vectors [13]–[16]. The shRNAs which are expressed by these vectors in the nucleus reach the cytoplasm using exportin-5, where they are processed by Dicer to the corresponding siRNAs [17]. In the context of antiviral therapy, an advantageous feature of RNAi is its reliance upon sequence complementarity, which confers the ability to discriminate the virus from host. This has led to experimental evaluation of RNAi, both *in vitro* and *in vivo*, as an antiviral tool against a very wide spectrum of viruses [8], [18], [19] and RNAi-based antiviral therapies against respiratory syncytial virus and human immunodeficiency virus are already being tested in the clinic [9]. In contrast, RNAi approaches targeting flaviviruses, particularly DENVs, are in early stages and focus on two aspects. One is to develop DENV resistance in mosquitoes, with the objective of blocking disease transmission [20]–[22], and the second is to inhibit DENV replication in mammalian cells using siRNAs targeting the envelope [23] and the NTRs [15], [24].

In this work, we have developed a replication-defective recombinant adenovirus (rAd) vector, rAdsh-5b, engineered to express a siRNA targeting a 5′NTR sequence element conserved across multiple DENV serotypes. We show that this vector is able to suppress efficiently the replication of genomic DENV RNA, the secretion of NS1 antigen, the release of DENV into the culture supernatant and the formation of DENV plaques. Interestingly, our data show that all four serotypes of DENVs are susceptible to RNAi mediated by the rAdsh-5b vector.

## Methods

### Cells and viruses

*E. coli* strains DH5α (for routine cloning) and BJ5183 (for rAd plasmid creation) were from Invitrogen. The prototypical representatives of the four DENV serotypes used in this study were (the strain and accession numbers are indicated in parantheses): DENV-1 (Nauru Island, U88535); DENV-2 (New Guinea C, AF038403), DENV-3 (H87, M93130) and DENV-4 (Dominica, M14931). The AdV5-transformed human embryonic kidney (HEK) cell line 293 and the monkey kidney Vero cell line were from American Type Culture Collection, Virginia, U.S.A. Cell lines were maintained in Dulbecco's Modified Eagle medium (DMEM), supplemented with 10% (v/v) fetal calf serum (FCS), in a 5% CO_2_ humidified incubator, at 37°C.

### shRNA plasmid construction

**Figure 1:**
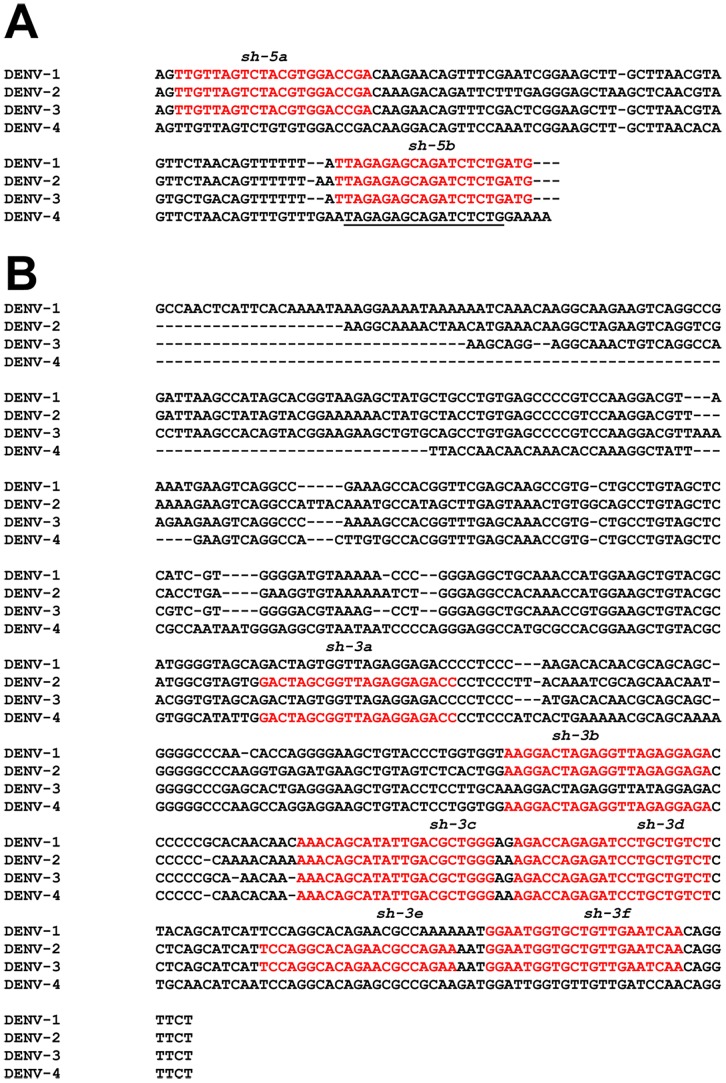
siRNA targets in the DENV NTRs. A ClustalW2 multiple alignment of 5′ (A) and 3′ (B) NTR sequences of the prototypic representatives of DENV-1, -2, -3 and -4 (described in Methods) showing the sites conserved in two or more serotypes targeted for RNAi in this study. NTR sequences that were utilized to design the sense strand of the sh constructs are shown in red fonts. The names of the sh constructs are shown in italics above the sequences in red fonts. The DENV-4 5′NTR seed sequence identical to the sh-5b target site is underlined.

Double-stranded 58-mer oligonucleotides encoding shRNAs targeting each of the sites indicated in Figure 1 were inserted in place of the 1.9 Kb stuffer sequence of pLKO.1 TRC vector [25], between the unique *Age* I and *Eco* RI sites, under the transcriptional control of the human U6 promoter as described in the Addgene plasmid repository website (http://www.addgene.org/10879/). The panel of constructs generated thus are listed in the first column of Table 1. The shRNA-encoding inserts in all constructs were verified by sequence analysis.

**Table 1:** The effect of NTR-specific shRNAs on DENV replication^a^.

shRNA Construct	Target site^b^	Conserved in serotypes	*Cis*-acting element^c^	Percent inhibition^d^			
				D-1	D-2	D-3	D-4
Sh-5a	3–23	1, 2, 3	SLA	33.5	5.1	0	29.1
Sh-5b	79–99	1, 2, 3	5′ UAR	51.3	48.2	63.8	70.8
Sh-3a	10,495–10,515	2, 4	nr	0	62.7	0	48.7
Sh-3b	10,579–10,600	1, 2, 4	nr	25.2	71.4	0	86.8
Sh-3c	10,616–10,636	1, 2, 3, 4	3′ CS	24.6	78.9	0	76.8
Sh-3d	10,639–10,659	1, 2, 3, 4	3′ UAR	16.6	92.6	0	80.3
Sh-3e	10,672–10,692	2, 3	3′ SL	19.5	78.7	0	81.1
Sh-3f	10,696–10,716	1, 2, 3	3′ SL	22	80.4	0	58.6

a The production of NS1 antigen, measured using BioRad's Platelia assay kit, served as a marker of DENV replication; data shown are from one representative screening experiment.

b This indicates the location of the 21 nts corresponding to the sense strand of the sh construct-encoded siRNA, on the DENV genome; numbers indicated correspond to DENV-2 NGC strain (Accession number AF038403).

c SLA: stem-loop A; 5′ UAR: 5′ upstream AUG region; 3′ conserved sequence; 3′ UAR: 3′ upstream AUG region; 3′ SL: 3′ stem-loop; nr: not reported.

d The percent inhibition was calculated with reference to DENV infectivity (in the presence of transfected sh-scr construct), which was taken as 100%. D-1, D-2, D-3 and D-4 denote DENV-1, DENV-2, DENV-3 and DENV-4, respectively.

### Screening of shRNA encoding plasmids

Vero cells, seeded in 96 well plates (at 6000 cells/well), two days in advance, were transfected with the shRNA plasmids listed in Table 1 using lipofectamine 2000 (Invitrogen). The lipofection mix (50 µl/well) contained 0.5 µg plasmid and 0.5 µl lipofectamine 2000 prepared in DME lacking serum and antibiotics as per the manufacturer's protocol. An equal volume of DME+10% heat-treated FCS (ΔFCS) was added 5 hours later. At 24 hours post transfection, the media was aspirated and the cells were infected with each of the four DENV serotypes at a multiplicity of infection (m.o.i) of 0.1–0.2 (in 50 µl DME+2%ΔFCS/well), separately. At 2 hours post-infection, each well received 200 µl DME+5%ΔFCS. Each transfection/infection was set up in triplicates and the cells were maintained at 37°C in 10% CO_2_ throughout. Four days later, suitably diluted aliquots of the culture supernatants were analysed for the presence of NS1 antigen using a commercially available ELISA kit (see below). Experiments were performed twice independently.

### Construction of rAds encoding shRNAs

The human U6 promoter-driven shRNA expression cassette was retrieved from selected shRNA constructs (sh-5b and sh-scr) above and inserted into the E1 region of the AdV5 genome of plasmid pAdEasy-1 by *in vivo* recombination in *E. coli* BJ5183 [26]. The resultant recombinant adenoviral plasmid was digested with *Pac* I to eliminate plasmid sequences and transfected into HEK 293 cells, to rescue the shRNA-encoding rAd viruses. Three rAds, one harboring the sh-5b cassette (rAdsh-5b), the next one harboring the sh-scr cassette (rAdsh-scr), and the last one harboring an insert-less hU6 cassette (rAd-Empty), were created for this study. They were verified by PCR and restriction analyses. All rAds were amplified and titrated on HEK 293 cells as described before [27]. To detect siRNA in rAd-infected cells, an RNase protection assay was performed using a commercially purchased kit (mirVana, Ambion Inc). Briefly, Vero cells were infected with rAdsh-5b (followed by DENV as described below) and total RNA prepared at different times post-infection. Total RNA (∼1 µg) was hybridized in solution to ∼50,000 cpm [P^32^]-radiolabeled sh-5b-specific sense (5′-TTAGAGAGCAGATCTCTGATGTTTTCCTGTCTC-3′) and antisense (5′-CATCAGAGATCTGCTCTCTAACCTGTCTC-3′) probes in 20 µl. After 4–5 hours the hybridized products were digested with the kit-provided RNase A/T1 cocktail (1∶100 diluted, 150 µl), ethanol precipitated and analysed on denaturing (8 M urea) 15% polyacrylamide gels. The radioactive bands were visualized using the Typhoon 9210 Imager (Amersham Biosciences).

### Vero cell infection

Vero cells were seeded in 12-well plates (10^5^ cells/well in 1 ml DME+10%ΔFCS) and incubated for 24 hours (37°C, 5% CO_2_). These were infected with either rAdsh-5b or rAdsh-scr (diluted in DME+2%ΔFCS), each at a m.o.i of 5 (0.25 ml/well). One entire plate of 12 wells was used for each rAd infection. After 1 hour adsorption 0.75 ml DME+5%ΔFCS was added to each well and the plate returned to the incubator. After 24 hours of exposure to rAd infection, the cells were subjected to DENV infection. For this, the culture supernatants from the wells were aspirated and the monolayers infected with 1000 PFUs of DENV-1, DENV-2, DENV-3 or DENV-4 in DME+2%ΔFCS (0.25 ml/well; 3 wells for each DENV serotype). Two hours later each well received 2.25 ml DME+5%ΔFCS. Plates were placed in the incubator for 1 week. Mock-infections (neither rAd nor DENV infected) and DENV infections (no rAd pre-infection) were set up in parallel. Aliquots (100 µl) of the culture supernatant were withdrawn at periodic intervals for analysis of DENV NS1 antigen, DENV genomic RNA and infectious DENV titers. In some experiments, instead of adding 2.25 ml DME+5%ΔFCS after DENV exposure, the wells were overlaid with methylcellulose containing medium to develop plaques (see below). Experiments were also performed wherein the order of infections was reversed with DENV infections first, followed 24 hours later with rAd infections. All infection experiments were performed twice.

### DENV NS1 determination

Culture supernatants collected at various time points, which were stored frozen at -20°C, were thawed and diluted 1∶10 using DME+2%ΔFCS. Suitable aliquots of this were used to detect DENV NS1 antigen using the commercially available Platelia Dengue NS1 kit (BioRad Inc), as per the manufacturer's protocol. This kit uses a pair of NS1-specific monoclonal antibodies to detect the NS1 antigen produced by all four DENV serotypes.

### RNA extraction, strand-specific cDNA synthesis and real time PCR

DENV RNA from infected culture supernatants (140 µl each) was extracted using the commercially available QIAmp viral RNA mini kit (Qiagen) as per the manufacturer's protocol. Total cellular RNA from mock-infected and DENV-infected cells was extracted using the Trizol reagent. The extracted RNA was dissolved in RNase-free water, quantitated by UV absorbance at 260 nm and stored frozen at −80°C until use. The RNA samples (250–500 ng total cellular RNA or 5 µl viral genomic RNA from culture supernatant) were used to synthesize cDNA using iScript Select cDNA synthesis kit (BioRad) according to the manufacturer's protocol. cDNA corresponding to the plus sense viral RNA (isolated from culture supernatant) was synthesized using primer D2. Each sample of cellular RNA was used to generate three cDNAs corresponding to the sense DENV, antisense DENV and GAP RNAs using primers D2, D1 and G2 (5′-AGGGGTCTACATGGCAACTG-3′), respectively. D1 and D2 are consensus primers common to the genomes of all four DENV serotypes, described earlier [28]. The cDNAs were used in real time PCR as follows. Each reaction contained 2.5 µl 10× SYBR green master mix, 5 µl of suitably diluted cDNA (1∶10, 1∶50 and 1∶100 dilutions were tested) and 12.5 pmoles of forward and reverse primers and water in a total volume of 25 µl. For both sense and antisense DENV cDNAs the primers were D1 and D2, and for the GAP cDNA, the primers were G1 (5′-CGACCACTTTGTCAAGCTCA-3′) and G2. Real time PCR was carried out using a BioRad MiniOpticon thermal cycler. A melt curve analysis was performed to ensure that amplification was specific. Both sense and antisense DENV RNAs in total cellular RNA were normalized to GAP RNA levels.

### Plaque assay

Vero cells seeded in 12 well plates (2×10^5^ cells/well/1 ml) were infected 24 hours later with 25 µl DENV-containing culture supernatants (collected at different time points in the Vero cell infection experiment above) in a final volume of 250 µl made up with DME+2%ΔFCS. Following 2 hours of adsorption, the inoculum was aspirated off and the monolayers overlaid with 1 ml 0.8% methyl cellulose prepared in 1× DME+6%ΔFCS and placed in the incubator (37°C; 10% CO_2_). Four days later viral plaques were developed using a modification of the immunostaining protocol described earlier [29] with a pan-DENV-specific monoclonal antibody, 4G2 [30].

### Statistical analysis

The statistical significance of differences between rAdsch-scr and rAdsh-5b treated samples (n = 6) was assessed using two-tailed Student's t test. Differences were considered statistically significant when the probability levels (P) were <0.05.

## Results

### A site in the 5′ NTR can be used to target all four DENV serotypes

As the DENV RNA genome contains a single ORF, theoretically one could target any site on it, either within the ORF or without, to achieve RNAi-mediated silencing effect. Several *cis*-acting elements in the NTRs flanking the ORF are important for successful completion of the viral life cycle (Figure S1). A 5′terminal stem-loop structure, SLA, functions as a promoter for ‘minus’ RNA synthesis [31]. Another stem-loop structure at the 3′ end (3′SL), is essential for RNA replication and virus viability [32]. Further upstream of the 3′SL is a conserved sequence (3′CS) involved in long-range interactions with a complementary sequence at the 5′end of the genome, 5′CS embedded in the C-encoding region [3]. Two more sequence elements, one in the 5′NTR, an upstream AUG region (5′UAR), and its complementary sequence (3′UAR) in the 3′NTR also contribute to long-range interactions between the two ends of the genomic RNA [33]. These long-range interactions mediate circularization of the viral genome, an event that is critical in both replication and translation [1]. In principle, targeting these NTR elements to RNAi may help achieve inhibition of viral replication. Consistent with their important roles in the viral life cycle, there is a high degree of conservation of these sequence elements among the DENV serotypes. We analysed the genomes of representative members of the four DENV serotypes for 21 nt uninterrupted stretches that may overlap with the *cis*-acting sequence elements of the NTRs, and may serve as putative siRNA target sites. Figure 1 presents a schematic representation of the putative siRNA target sites chosen for this study. We identified two sites in the 5′NTR (sh-5a and sh-5b) that overlapped with SLA and 5′UAR (Figure S1), respectively, and were completely conserved in DENV-1, -2 and -3. In the 3′ NTR, we identified six potential sites, two (sh-3c and sh-3d), mapping to the 3′CS and 3′UAR, were conserved across all 4 DENV serotypes, while the remaining were conserved across either two or three serotypes. Of these, two sites (sh-3e and sh-3f) mapped to the 3′SL. Using these conserved sequences as putative siRNA target sites, we next created a panel of shRNA-encoding plasmid constructs. These shRNA constructs and the sites targeted by them on the DENV genome, are shown in Table 1 (see also Figure S1). Each of these constructs was screened against each one of the four DENV serotypes as explained below.

Vero cells were first transfected with the different shRNA constructs, including a control construct encoding a scrambled shRNA insert (sh-scr), and infected 24 hours later with each one of the four DENVs separately. We then assessed the levels of DENV replication using NS1 antigen secretion as a surrogate marker. These data are also shown in Table 1, which summarizes the percent inhibition of the DENVs by the different shRNA constructs with reference to DENV infectivity in cells pre-transfected with sh-scr plasmid taken as 100%. The data indicate that all shRNAs are successfully processed to their cognate siRNAs as evidenced by their ability to inhibit replication of one or more DENV serotypes. However, the observed levels of inhibition varied widely. Two constructs, sh-3a and sh-3b, behaved predictably in that they inhibited the DENV serotypes they were designed to target. The remaining manifested DENV inhibitory profiles seemingly at odds with their design. For example, sh-3c and sh-3d, targeting the 3′CS and 3′UAR elements, respectively, are conserved in all four DENV serotypes. Yet, DENV-3 was unaffected by these sh constructs. Similarly, sh-3e and sh-3f targeting the 3′SL are fully conserved in DENV-2 and -3, and DENV-1, -2 and -3, respectively. But these constructs were most effective against DENV-2, with no efficacy whatsoever against DENV-3. The apparent resistance of DENV-3 to RNAi-mediated inhibition by the panel of shRNAs tested suggests key differences in the overall secondary structure of the DENV-3 NTRs. The construct sh-3e showed marginal efficacy against DENV-1. DENV-4 was susceptible to inhibition by sh-5b, sh-3e and sh-3f, despite multiple nt mismatches in the sequence stretches in its NTRs corresponding to these constructs. This may be due to microRNA (miRNA)-like action, a suggestion consistent with the view that siRNAs can function as miRNAs when complementarity with the target site is restricted [9], [34]. The notable finding in this screening experiment was the identification of a single construct, sh-5b, which inhibited all four DENV serotypes (48–71%). Interestingly, the target site of sh-5b, which encompasses 5′UAR, is fully conserved in DENV serotypes 1–3. Further, a continuous stretch of 19 internal nts (nt #s 2-18) of the sh-5b target site, encompassing its seed sequence, is also completely conserved in DENV-4. We analysed the 5′NTRs of the first dozen DENV isolates from different geographical regions, corresponding to the four serotypes, listed in the dengue virus portal of Broad Institute and found the sh-5b target site to be highly conserved (Figure S2). Based on the results presented above, we decided to proceed with the sh-5b construct for further work.

### Recombinant adenovirus-mediated expression of sh-5b siRNA

**Figure 2:**
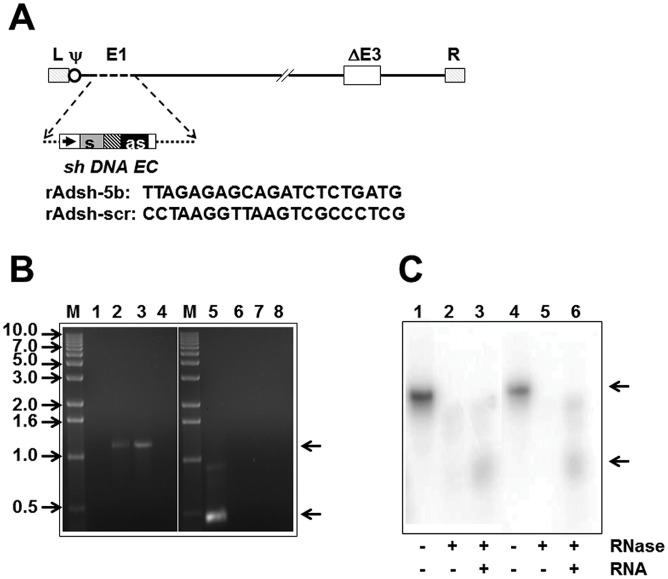
Design and characterization of the rAd-sh viruses. (A) The linear genome of the rAd-sh virus constructed for this study. In constructing the rAd-sh virus, the E1 region (dashed line) is replaced by the sh DNA expression cassette (*sh DNA EC*), consisting of the U6 promoter (rightward arrow), the shDNA insert with the sense (s) and antisense (as) arms, of 21 base pairs each, followed by of the U6 terminator (empty box). The shaded box between the ‘s’ and ‘as’ arms is a 6-base pair loop sequence. The dotted lines flanking the expression cassette represent plasmid vector sequences. Other elements of the rAd-sh genome include a ∼2.7 Kb deletion in the E3 region (ΔE3), the left (L) and right (R) inverted terminal repeats, and the packaging signal (ψ). The nt sequences of the ‘s’ strands of the sh-5b and sh-scr constructs are shown below. (B) PCR analysis of wild type AdV5 (lanes 1 & 5), rAdsh-E (lanes 4 & 8), rAdsh-5b (lanes 2 & 6) and rAdsh-scr (lanes 3 & 7) using insert-specific (lanes 1–4) and AdV5 E1-specific (lanes 5–8) primers. DNA size markers (sizes in kb shown to the left) were analyzed in lanes ‘M’. The arrows to the right denote the positions of the predicted insert-specific (upper) and AdV5 E1 region-specific (lower) amplicons. (C) RNase protection assay to detect anti-sense strand of sh-5b siRNA. A radiolabeled sense probe was digested with RNases A and T1, either before (lanes 2 & 5) or after hybridization with total RNA isolated from rAdsh-5b-infected Vero cells, harvested on either day 3 (lane 3) or day 8 (lane 6) post-infection. Protected fragments (lower arrow) were analysed on 8 M urea gel and visualized using a phosphoimager. The un-hybridized probe (upper arrow) without any RNase treatment was analysed in parallel (lanes 1 & 4). It is to be noted that in lanes 3 and 6, the cells used for total RNA preparation were challenged with DENV-2 and DENV-4, respectively, at 24 hours post rAdsh-5b infection.

As the introduction of shRNA through a plasmid transfection approach would be impractical for future use in an *in vivo* setting, we created a vector based on AdV5, whose potential to deliver shRNA successfully has been documented [13], [14]. Further, it has been shown that the endogenous RNAi and interferon pathways are not significantly affected in cells infected with a recombinant AdV5 vector encoding sh-RNA [35]. A fragment containing the human U6 promoter-driven shRNA expression cassette, retrieved from the sh-5b plasmid, was inserted into the early region 1 (E1) of AdV5 genome using established strategy [26], [27]. A schematic representation of this rAd vector, rAdsh-5b, is shown in Figure 2A. In parallel, we also created two additional control rAd vectors, rAdsh-scr, encoding a scrambled shRNA expression cassette and, rAdsh-E, containing an empty (insertless) U6 cassette. The replacement of the E1 region by the ‘sh’ insert in the genomes of these rAd viruses was verified by PCR analysis using insert-specific and E1-specific primers [27]. The results, in Figure 2B, show that the use of the insert-specific primers generated the predicted ∼1.2 kb amplicons from the genomes of rAdsh-5b and rAdsh-scr (lanes 2 & 3), but not from those of wild-type (wt) AdV5 and rAdsh-E (lanes 1 & 4). Similarly, the use of E1 primers produced the expected ∼0.47 kb amplicon only when the template was wt AdV5 DNA (lane 5), but not when it was from any of the three rAdsh viruses (lanes 6–8). These data were corroborated by extensive restriction analyses as well (data not shown).

Vector-borne shRNAs which are transcribed in the nucleus are similar to pre-microRNAs and like them, mature using the same Exportin-5/Dicer pathway to siRNAs found in the cytoplasm [17]. To ascertain if the rAdsh-5b vector gives rise to the corresponding sh-5b siRNA *in vivo*, we analysed Vero cells infected with this vector at various times post-infection using an RNase protection assay. Total RNA from rAdsh-5b infected cells was hybridized to a 33 nt long radiolabeled sense probe, subjected to RNase treatment and analysed on denaturing gels to detect the 21 nt protected antisense strand of sh-5b siRNA. Results from a typical experiment performed with total RNA obtained at two different time points are shown in Figure 2C. The sense probe was specifically protected in the presence (lanes 3 & 6), but not in the absence (lanes 2 & 5) of total RNA derived from cells that had been exposed to rAdsh-5b infection, indicating the presence of the complementary antisense strand of sh-5b siRNA. Interestingly, we could detect the antisense strand as late as eight days following rAdsh-5b infection. This was corroborated by detection of the sense strand of this siRNA using a complementary antisense probe (data not shown). These data confirmed the successful intracellular delivery of siRNA by the rAd vector.

### The rAd vector *per se* does not affect replication of any of the DENV serotypes

Before analysing the effects of rAdsh-5b mediated RNAi on DENV replication, we sought to ascertain that the rAd vector does not in itself interfere with DENV replication. In order to do this, we compared the DENV genomic RNA levels in cells in the presence and absence of rAdsh-scr control vector, encoding an irrelevant scrambled shRNA, referred to above. In this experiment, Vero cells were first infected with rAdsh-scr, followed 24 hours later with DENV-1, DENV-2, DENV-3 or DENV-4. Viral sense RNA isolated from the culture supernatant at different times post-DENV infection was analyzed by real time RNA PCR. We examined the levels of both sense and antisense viral RNAs in total cellular RNA prepared at different times post-DENV infection (Figure S3). We found that both kinds of RNA samples, obtained either from cells infected only with DENV or from those pre-infected with rAdsh-scr prior to DENV infection, manifested virtually similar real time amplification profiles, for all four DENV serotypes (p>0.05). Further, this was true regardless of whether we examined viral RNA in culture supernatant or total cellular RNA, on day 2 or day 7 post-DENV infection. These data demonstrated that neither the sh-scr RNA construct nor the rAd vector used to deliver it into cells had any discernible effect on DENV replication. Thus, the rAdsh-scr vector could serve as a good control to evaluate the effects, if any, induced by the rAdsh-5b vector on DENV replication.

### sh-5b siRNA delivered using rAd vector is a powerful inhibitor of DENV-1, -2, -3 and -4

**Figure 3:**
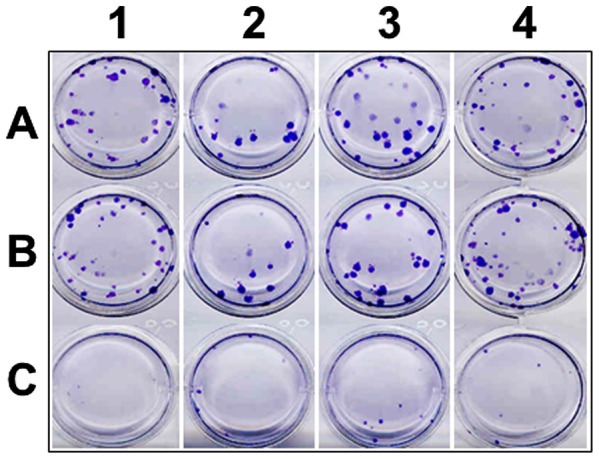
The effect of rAd mediated sh RNA expression on DENV plaque formation. Vero cells in 12-well plates, were sequentially infected with rAdsh-5b (row C) or rAdsh-scr (row B), and 24 hours later, with DENV-1 (column 1), DENV-2 (column 2), DENV-3 (column 3) and DENV-4 (column 4). In parallel control experiments, the rAd infection was omitted and only DENV infections performed (row A). Cells were overlaid with methyl cellulose and stained four days later with a pan-DENV-specific monoclonal antibody to visualize the viral plaques. One experiment of two performed is shown.

Next, using rAdsh-scr as the reference, we evaluated the effect of rAdsh-5b on prototypic representatives of all four DENV serotypes in a plaque assay format. In this experiment, Vero cells were first infected with either rAdsh-scr (control) or rAdsh-5b (test) and then challenged 24 hours later with each of the DENV serotypes separately. Immediately following DENV infection, the monolayers were overlaid with methyl cellulose and incubated to allow plaque formation. The results are shown in Figure 3. This experiment shows that rAdsh-scr pre-infection had no discernible effect on the number of DENV plaques formed in all four serotypes (compare rows A & B), In striking contrast, pre-treatment with rAdsh-5b resulted in a remarkable level of suppression of plaque formation by all four DENV serotypes (row C). This observation which is consistent with the behaviour of the plasmid-borne sh-5b construct in the screening experiment described above demonstrates the feasibility of achieving inhibition of all four DENV serotypes using a rAd vector to deliver sh-5b siRNA into cells. However, as plaque reduction assays are usually performed in 24-well plates with limited amount of DENV (50 PFUs/well), it is likely that this may be responsible for the near total inhibition of the DENVs in this experiment.

### Significant DENV inhibition can be achieved by rAdsh-5b at high DENV dosage

We next sought to address if the rAdsh-5b would be effective in inhibiting DENVs if the challenge dose were to be significantly increased. To this end, we set up an experiment similar to the one above with the following two differences. One, we increased the dose of challenge DENV 20-fold (1000 PFUs/well); and two, instead of methyl cellulose overlay, liquid growth medium was added to cells followed by sampling of cultures periodically for 1 week post-DENV infection for analysis of genomic RNA levels, NS1 antigen secretion, and infectious virus production.

**Figure 4:**
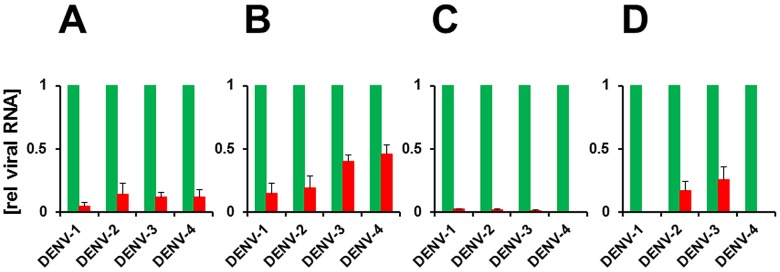
The effect of rAd-mediated shRNA expression on DENV RNA accumulation. Vero cells were pre-infected either with rAdsh-5b (red bars) or rAdsh-scr (green bars) followed 24 hours later by infection with DENV-1, DENV-2, DENV-3 and DENV-4. Total RNA was isolated on days 2 (panels A and B) and 7 (panels C and D) post-DENV infection and analyzed for DENV ’minus’ (panels A and C) and ‘plus’ (panels B and d D) sense viral genomic RNAs by strand-specific real time PCR analyses. DENV RNA was normalized to GAP RNA in each sample analyzed. The data depict DENV RNA levels in rAdsh-5b treated cells relative to those in the corresponding rAdsh-scr treated cells. Each experiment was carried out in triplicate wells and the entire experiment repeated twice.

The results of a comparison based on real time RNA PCR assay of the relative DENV genomic RNA levels (normalized to GAP RNA levels) in DENV-infected cells between the rAdsh-scr pre-treated (control) and rAdsh-5b-pre-treated (test) samples are summarized in Figure 4. In this experiment the DENV RNA level in the test sample has been expressed relative to that in the corresponding control sample, which was arbitrarily set at 1.0. It is immediately apparent that test RNA levels are a fraction of the control RNA levels across the board, showing that rAdsh-5b exerted a uniformly inhibitory effect on all DENV serotypes. An analysis of sense viral RNA levels at day 2 post DENV infection is presented in panel B. DENV-1 and -2 sense RNA levels manifested very high inhibition (>80%) compared to their corresponding controls, while DENV-3 and DENV-4 sense RNA levels suffered relatively lesser degree of reduction (∼50–60%). By day 7, sense RNA was virtually indiscernible for DENV-1 and -4, and >70% reduced in DENV-3, with respect to their cognate controls. DENV-2 sense RNA levels did not manifest further reduction beyond ∼80% seen at day 2 post-DENV infection (panel D). The observed inhibition in the levels of sense viral RNA seen for all four DENV serotypes was mirrored in the antisense RNA levels as well (panels A and C), with the inhibition being relatively greater than that observed for sense RNA. Further, the reduction in antisense RNA levels was more pronounced on day 7 (panel C) compared to that on day 2 (panel A), as seen for sense RNA levels. The greater susceptibility of antisense DENV RNA to inhibition, compared to its sense counterpart, may be a reflection of the lower copy number of the former [3]. In all instances, the reduction in genomic RNA of the test samples, compared to the cognate reference samples, was significant (P<0.05).

**Figure 5:**
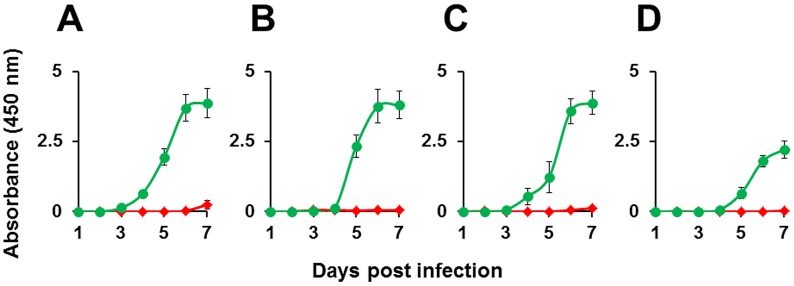
The effect of rAd-mediated shRNA expression on DENV NS1 antigen secretion. Vero cells were pre-infected either with rAdsh-5b (red curves) or rAdsh-scr (green curves) followed 24 hours later by infection with DENV-1 (A), DENV-2 (B), DENV-3 (C) and DENV-4 (D). Culture supernatants were drawn at daily intervals up to 7 days post DENV infection and analyzed for the presence of NS1 antigen using BioRad's Platelia Dengue NS1ELISA kit. The data represent plots of NS1 ELISA absorbance as a function of time after DENV infection. Data shown are mean values (n = 6). The vertical bars represent standard deviation, SD.

During the course of the experiment described above, we also withdrew aliquots of the culture supernatant at regular intervals and determined the levels of NS1 antigen using a commercially available ELISA kit. These data are depicted in Figure 5. In the control samples, NS1 secretion was discernible after day 3 post-DENV infection, which peaked about 2–3 days later and plateaued thereafter. While the NS1 secretion profiles of DENV-1, -2 and -3 were more or less comparable to each other, the NS1 secretion profile of DENV-4, though qualitatively similar, was quantitatively lower in the controls. This is presumably inherent to DENV-4 or a reflection of the lowered sensitivity of the kit in picking up DENV-4 NS1. The kinetics of NS1 secretion by the DENVs in the absence of prior rAdsh-scr infection was essentially similar (data not shown). Remarkably, we found that NS1 antigen secretion was almost completely inhibited in the test samples compared to their respective controls, for all four DENV serotypes tested (P<0.05).

**Figure 6:**
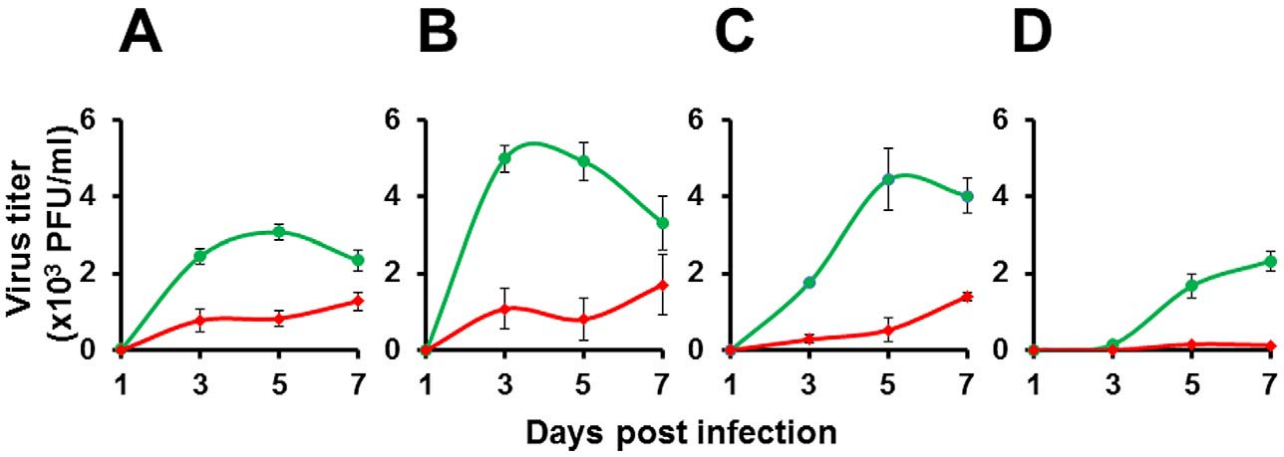
The effect of rAd mediated shRNA expression on DENV secretion. Vero cells were pre-infected either with rAdsh-5b (red curves) or rAdsh-scr (green curves) followed 24 hours later by infection with DENV-1 (A), DENV-2 (B), DENV-3 (C) and DENV-4 (D). Culture supernatants were drawn at daily intervals up to 7 days post DENV infection and analyzed for the presence infectious DENV using a standard plaque assay. Data shown are mean values (n = 6). The vertical bars represent SD.

The suppression in viral antigen secretion would suggest that virus production would also be reduced. This was borne out by an examination of the virus titers in the culture supernatant. Figure 6 depicts virus titers, measured using a standard plaque assay, in the same aliquots used for NS1 determination described above. The virus titers in the case of DENV-1, -2 and -3 controls rose gradually, peaking at day 5 post-DENV infection. The DENV-4 control experiment manifested somewhat delayed kinetics, peaking at day 7, a trend mirroring the NS1 secretion profile described above. In comparison, the rate of virus secretion into medium in the test experiments was noticeably, but not completely, ablated. This contrasts with the observed NS1 secretion profiles (Figure 5), which manifested near total ablation in response to rAdsh-5b pre-treatment. Observed DENV titers in the test groups manifested 70–90% reduction compared to their corresponding controls (P<0.05).

### rAdsh-5b is effective against on-going DENV infection

**Figure 7:**
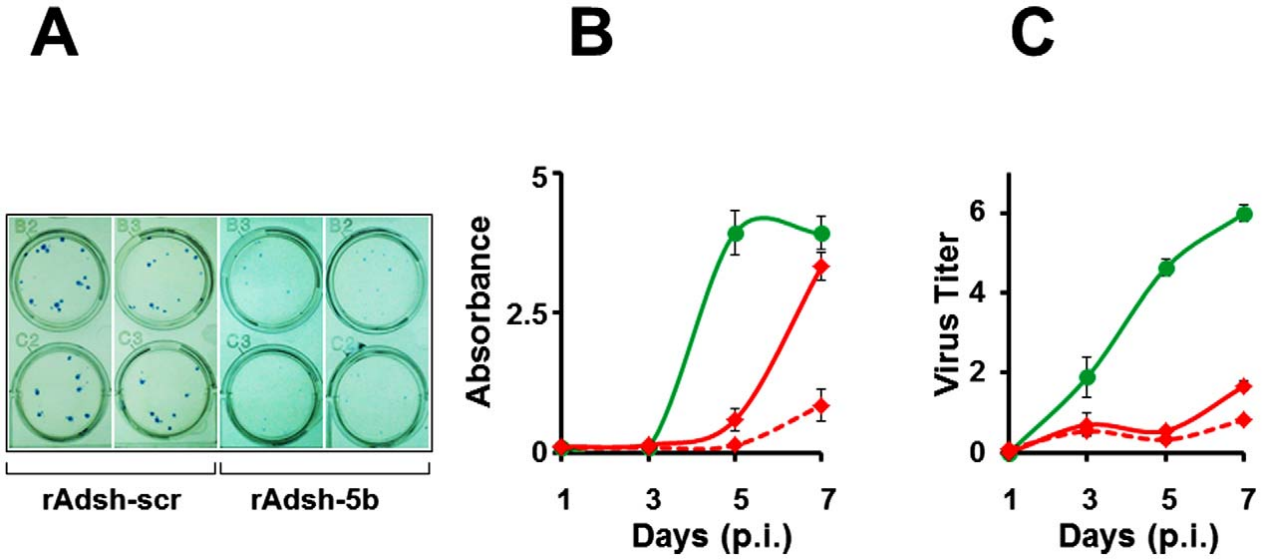
The effect of rAd mediated shRNA expression on on-going DENV infection. (A) Vero cells in 12-well plates were sequentially infected with DENV-2 (∼25 PFU/well) and 24 hours later, with rAdsh-scr or rAdsh-5b, each at a m.o.i of 5 (top row) or 10 (bottom row). Cells were overlaid with methyl cellulose and plaques visualized as explained in Figure 3 legend. Two wells, of four assayed for each sequential infection experiment, are shown. (B) Vero cells in 24-well plates were sequentially infected with DENV-2 (1000 PFUs/well), followed 24 hours later with rAdsh-5b (red curves) or rAdsh-scr (green curve), each at m.o.i. of 5 (solid curve) or 10 (dashed curve). Culture supernatants were drawn at 48 hour intervals up to 7 days post DENV infection and analyzed for the presence of NS1 antigen using BioRad's Platelia Dengue NS1ELISA kit. The data represent plots of NS1 ELISA absorbance as a function of time after DENV infection. Data shown are mean values (n = 4). The vertical bars represent SD. (C) Culture supernatants in (B) were analyzed for the presence infectious DENV using a standard plaque assay. Data shown are mean values (n = 4). The vertical bars represent SD.

In the experiments described so far, the sh-5b shRNA was already introduced into the cells before infecting them with DENV. To get a more accurate assessment of the possible therapeutic potential of this approach, we infected cells first with DENV and then introduced the shRNA *via* the rAd vector. In the first such experiment, which was a plaque reduction assay similar to the one in Figure 3, cells were infected first with DENV-2, and 24 hours later with either rAdsh-scr or rAdsh-5b, and immediately overlaid with methyl cellulose-containing medium. Two doses of each rAd virus were tested in this experiment, one corresponding to the dose in the earlier set of experiments (m.o.i = 5) and the other which was twice as much (m.o.i = 10). Typical results are presented in Figure 7A. It is evident that at both doses of rAdsh-5b tested, a noticeable reduction in plaque counts was discernible. Next, we repeated this experiment, as before, with a higher dose of DENV-2 (1000 PFU/well), followed 24 hours later with rAdsh-scr or rAdsh-5b, at two different doses as above. Infected cells were fed with liquid medium which was sampled at 48 hour intervals over a one-week period for analysis of NS1 antigen levels and DENV-2 titers. These data are summarized in Figures 7B and 7C, respectively. NS1 antigen secretion which was initially inhibited by rAdsh-5b, at the lower dose, reached control levels by day 7 post-infection. However, this could be counteracted at higher rAdsh-5b dosage (Figure 7B). In contrast, the lower dose of rAdsh-5b was nearly as effective as the higher dose in inhibiting the production of infectious DENV-2 (Figure 7C). The data in Figures 7B and 7C, taken together, would suggest that at the lower dose of rAdsh-5b tested, continued translation of the residual viral genomic RNA is responsible for the observed rise in NS1 levels (at day 7). This is consistent with the observation that increasing the rAdsh-5b dosage brings down the NS1 levels, presumably through increased degradation of the DENV-2 genomic RNA.

## Discussion

One approach to deploying RNAi against multiple DENV serotypes would be to target its action to conserved sequences on the genomes of the four DENV serotypes [15], [24]. In this report, we have explored the feasibility of targeting conserved *cis*-acting elements in the NTRs to achieve RNAi-mediated inhibition of multiple DENV serotypes. We tested each of these putative sites, on each DENV serotype, using plasmid-delivered shRNAs, by assaying the levels of NS1, a viral antigen used as a marker of virus replication [36], [37]. An earlier study which reported that a shRNA targeting the 3′ CS region can inhibit DENV-2 infection in Vero cells and DCs implied that it may be a pan-DENV inhibitor [15]. However, we found that the sh-3c construct (targeting 3′ CS) was active on DENV-2 and DENV-4, but not on DENV-1 and DENV-3. This, taken in conjunction with our results (Table 1) lead to the suggestion that an interplay of other factors, such as structural constraints and the degree of accessibility of the conserved target sequence presumably determine RNAi susceptibility. Our work which showed that sequence complementarity alone does not ensure susceptibility of all four DENV serotypes to RNAi, helped experimentally identify one construct, sh-5b, as a pan-DENV inhibitor. Interestingly, 21/21 sh-5b target site nts are fully conserved in DENVs 1–3, whereas in DENV-4, 19/21 nts are conserved. Significantly, all four DENV serotypes have an intact 5′UAR element, which is presumably accessible to the RNAi machinery.

Next, we developed an AdV5-based viral vector, rAdsh-5b, for its efficient intracellular delivery, after ensuring that the AdV5 vector *per se* did not affect DENV replication. We showed that AdV5 vector-delivered sh-5b shRNA matures into its cognate siRNA, which persisted for several days in rAdsh-5b-infected Vero cells. We analysed the effect of rAd-mediated delivery of sh-5b siRNA on the replication of all four DENV serotypes in terms of antigen synthesis, viral RNA synthesis and infectious virus production. In these experiments, cells pre-exposed to rAdsh-5b were challenged with different DENVs. While DENV NS1 antigen levels manifested near total suppression in rAdsh-5b pre-treated cells, viral RNA accumulation and infectious virus production were significantly, but not completely, abolished (Figures 3, 4, 5, 6). Importantly, we did not observe any discernible change in the levels of GAP RNA levels in response to rAdsh-5b treatment suggesting that the likelihood of possible off-target effects may be low. Interestingly, we observed that a DENV-2 infection which was already underway for 24 hours was also susceptible to RNAi induced by rAdsh-5b in a dose-dependent manner (Figure 7). Earlier studies have not examined this aspect [15], [24], and this work demonstrates for the first time that RNAi, mediated by an AdV5 vector, may have potential utility as an antiviral tool in the context of on-going DENV infection. A similar AdV5 vector-based shRNA approach has shown promise in attenuating pre-established HBV infection [14]. However, our results raise several issues as discussed below.

A recent study which screened a panel of DENV-specific siRNAs in Huh7 cells using plaque assays and Western blotting assays, found that one of them targeting the 5′CS element functioned as a pan-DENV inhibitor [24]. Though the present study did not include 5′ CS as a target for RNAi, the observation that the sh-3c construct (which targeted the complement of 5′CS in the 3′ NTR), inhibited DENV-2 and DENV-4, may be regarded to be partly consistent with the earlier report [24]. However, two additional siRNAs in the earlier study, DC-1 and DC-2, whose target sites happened to overlap considerably with the sh-5a and sh-5b sites, respectively, yielded results that were different from ours. Specifically, the DC-2 siRNA which shared 18 of the 21 nts comprising the sh-5b target site failed to inhibit any of the DENVs. The reason for the apparent differences between the previous and current studies is unclear. A definitive comparison is difficult given the differences in cell types, methodology, subtle differences between the siRNAs (especially at the 3′ends) and the mode of delivery in the two studies.

Regardless of whether rAdsh-5b was used before or after onset of DENV infection we did not achieve complete inhibition of virus replication. This is an inherent attribute of RNAi-mediated silencing and could be perceived as a limitation of this approach in a therapeutic context, wherein total inhibition is necessary. One may anticipate achieving higher levels of inhibition by targeting additional sites on the DENV genomes (see below). Also, increasing the dose of the shRNA-encoding vector may contribute to greater efficiency of silencing. However, in the case of dengue the difference between mild and potentially fatal disease is the high viral load in the latter [5], [6]. Based on this it may be speculated that a reduction in viral load may be adequate to attenuate disease severity.

We have tested one prototypic representative of each of the four DENV serotypes. Would multiple isolates within each of the four DENV serotypes manifest susceptibility to sh-5b siRNA? An analysis of the 5′NTR sequences of several isolates of DENV-2, and -3 serotypes listed in the Dengue Virus portal of the Broad Institute revealed absolute conservation of the sh-5b target site. In the case of DENV-1, Vietnamese isolates differed in one nt, but several Cambodian and Thai DENV-1 isolates listed in this database, once again displayed 100% conservation of the sh-5b target site (not shown). In the case of DENV-4, the isolates displayed 90% identity with sh-5b target sequence, with all possessing an intact 5′ UAR element, similar to the prototypic DENV-4 strain examined in this study. By and large, the sh-5b target sequence is highly conserved. The possibility of the inherently error-prone RNA replication machinery facilitating escape from RNA may be counteracted to a certain extent by the high degree of conservation of the sh-5b target site. However, our observation of residual viral RNA and infectious viral titers, especially at high DENV challenge doses, suggests that it would be desirable to develop vectors that could encode additional shRNAs, collectively targeting each of the DENV serotypes, preferably at more than one or two sites.

How appropriate is it to use AdV5 as a shRNA delivery vector, given its potential to interact with the host antiviral response pathways? AdV5-encoded virus-associated RNAs (VA-RNAs) have the potential to interact with RISC and interfere with the RNAi pathway [38], [39]. In this regard, it is relevant to note that our vectors lack E1 genes, a defect associated with extremely low levels of VA-RNA expression [38]. Further, experimental analysis of cells infected with a rAdsh-RNA vector has not revealed any significant effect on either the RNAi or the interferon response pathways [35]. This is consistent with our observation that the rAd vector *per se* does not affect DENV replication and emphasizes the utility of AdV5 as a suitable vector for mediating shRNAs into the cell. Another issue concerning the suitability of AdV5 vector is that its efficacy may be compromised in a majority of the human population which is seropositive for AdV5 [40]. However, it has been shown that AdV5 complexed to specific antibodies can be taken up into macrophages and dendritic cells through Fc receptors [40], [41]. As these are the very cell types wherein DENV replicates *in vivo* [42], [43], an intriguing possibility is that the existence of anti-AdV5 antibodies may in fact facilitate the delivery of the shRNAs into these cells.

In conclusion, the observation of suppression of all four DENV serotypes based on multiple parameters such as viral antigen secretion, genomic RNA levels and infectious virus production, which are mutually corroborative demonstrate the utility of rAd-mediated delivery of shRNAs as an effective tool to abrogate DENV replication. However, additional conserved and accessible sites need to be targeted to counter viral escape. We note that these results obtained using a tissue culture model need to be verified in a small animal model such as AG129. A recent study has achieved DENV inhibition in this model using chemically synthesized siRNA [24]. As the difference between severe and mild disease appears to be dependent on the viral load, early diagnosis can facilitate timely therapeutic RNAi-based intervention to change the course of disease from DHF to DF by bringing down the high viremia.

## Supporting Information

Figure S1 ***Cis*** **-acting DENV NTR elements targeted by the sh constructs.** The DNA sequences shown correspond to 5′ (A) and 3′ (B) NTRs of DENV-2 New Guinea C strain (Accession no. AF038403). The *cis*-acting sequence elements, SLA, 5′ UAR, 3′ CS and 3′ UAR are underlined. The 3′ SL element is double-underlined to delineate it from the 3′ UAR right next to it. Nts in red font denote the sequences targeted for RNAi, with the names of the corresponding sh constructs shown above in italics.
(TIF)

Figure S2 **Conservation of the sh-5b/5′ UAR sequences within and across the four DENV serotypes.** The figure displays ORF-proximal region of the 5′ NTRs of the first dozen isolates of each DENV serotype listed in the Broad Institute dengue virus portal (http://www.broadinstitute.org/annotation/viral/Dengue/SequenceSearch.html). Corresponding DNA sequences were retrieved from NCBI. The accession numbers of the isolates are shown on the left. The sh-5b target sequence is highlighted in yellow. The single nt mismatch in many of the DENV-1 isolates examined is highlighted in green. Shown on top is the sequence of the sh-5b target site, with the 5′ UAR sequence within it shown by the underline.
(TIF)

Figure S3 **Real time PCR analysis of DENV RNA levels in Vero cells pre-infected with rAdsh-scr.** Vero cells were either mock-pre-infected (blue curves) or pre-infected with rAdsh-scr (green curves) for 24 hours followed by infection with DENV-1 (A), DENV-2 (B), DENV-3 (C) and DENV-4 (D). Total cellular RNA was isolated on day 7 post-DENV infection and analyzed for DENV ‘plus’ sense genomic RNA by real time PCR. The horizontal dashed line indicates the baseline used to determine Ct values.
(TIF)
