# Supplementary figures and images for: Adenovirus Delivered Short Hairpin RNA Targeting a Conserved Site in the 5′ Non-Translated Region Inhibits All Four Serotypes of Dengue Viruses
Source: PLoS Negl Trop Dis. 2012 Jul 24;6(7):e1735. doi: 10.1371/journal.pntd.0001735 (PMC3404111; doi:10.1371/journal.pntd.0001735)

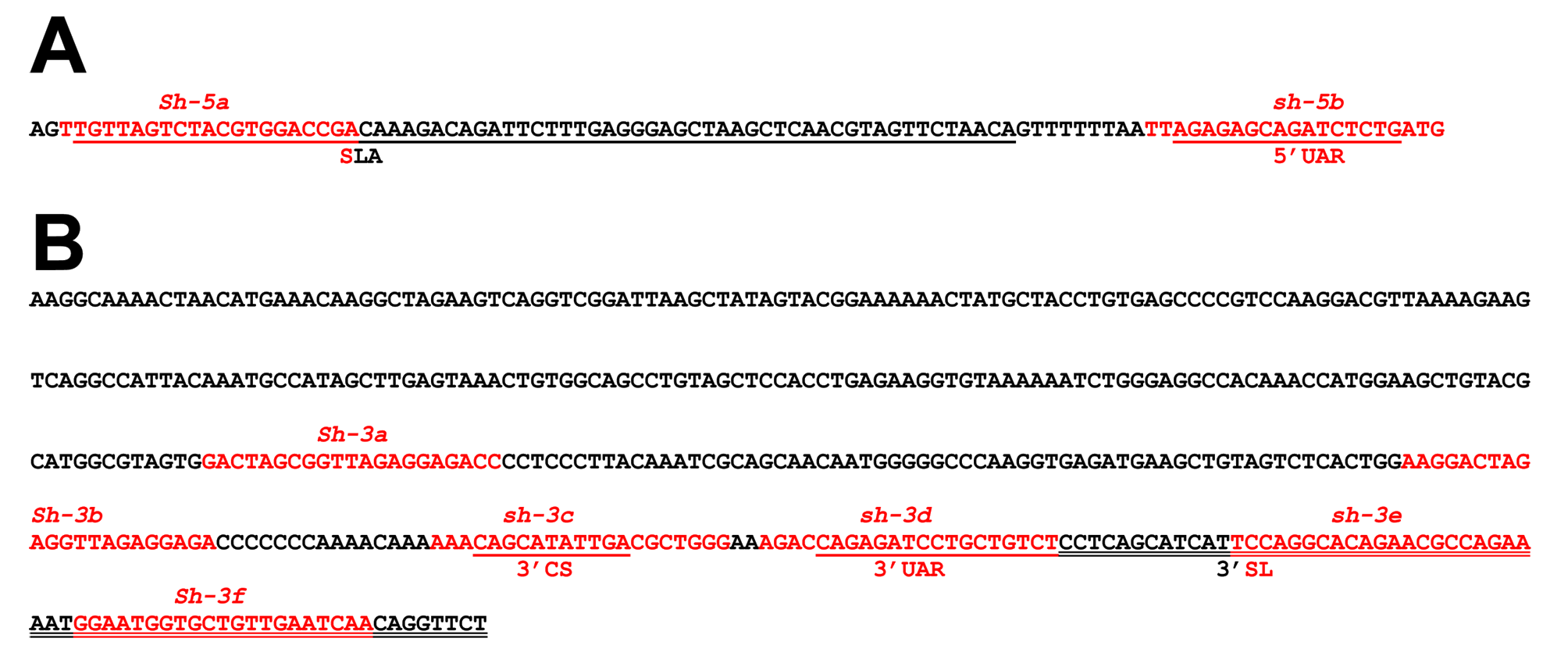

Supplement: Figure S1 — Cis -acting DENV NTR elements targeted by the sh constructs. The DNA sequences shown correspond to 5′ (A) and 3′ (B) NTRs of DENV-2 New Guinea C strain (Accession no. AF038403). The cis-acting sequence elements, SLA, 5′ UAR, 3′ CS and 3′ UAR are underlined. The 3′ SL element is double-underlined to delineate it from the 3′ UAR right next to it. Nts in red font denote the sequences targeted for RNAi, with the names of the corresponding sh constructs shown above in italics. (TIF) [file pntd.0001735.s001.tif]

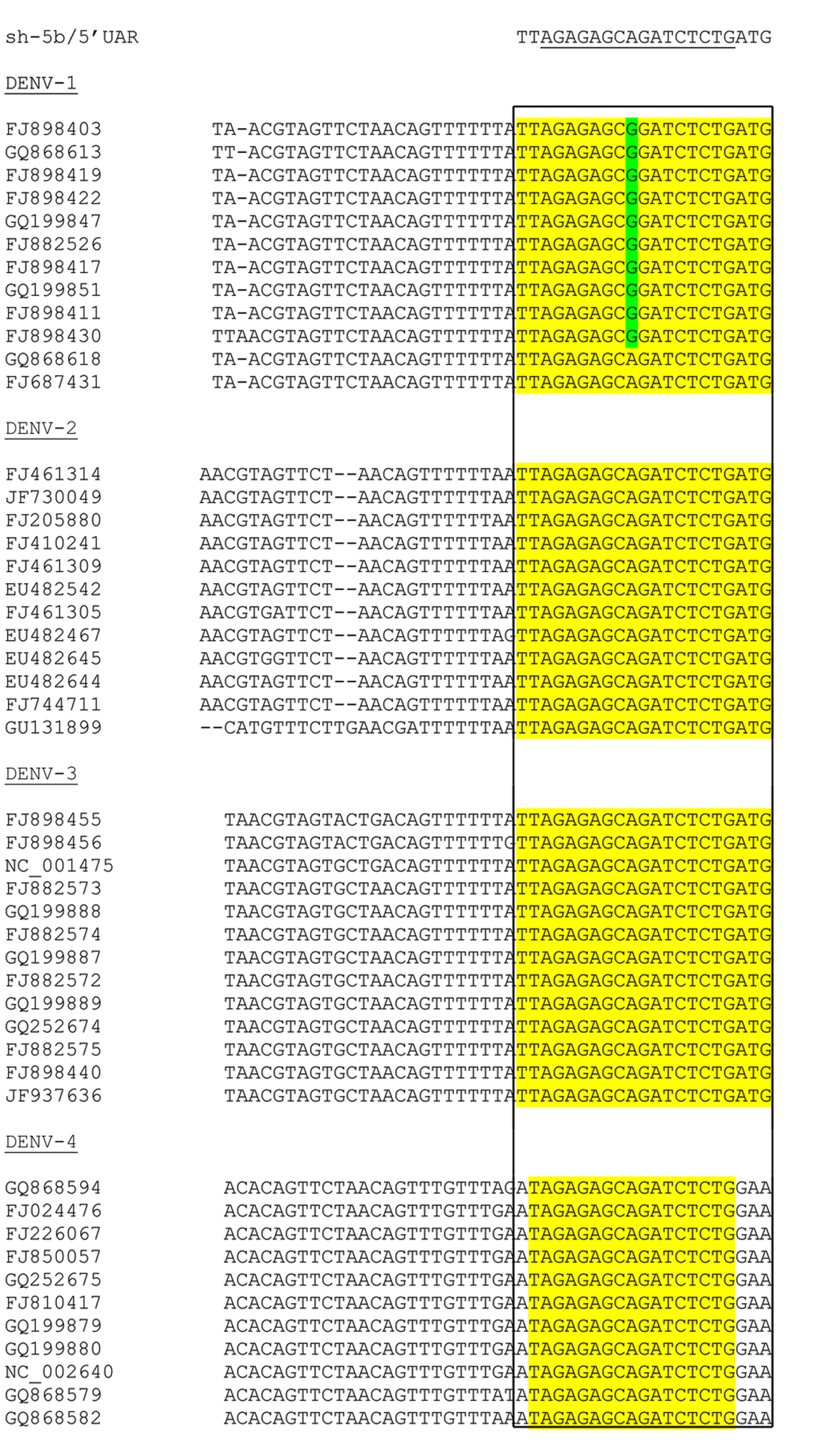

Supplement: Figure S2 — Conservation of the sh-5b/5′ UAR sequences within and across the four DENV serotypes. The figure displays ORF-proximal region of the 5′ NTRs of the first dozen isolates of each DENV serotype listed in the Broad Institute dengue virus portal (http://www.broadinstitute.org/annotation/viral/Dengue/SequenceSearch.html). Corresponding DNA sequences were retrieved from NCBI. The accession numbers of the isolates are shown on the left. The sh-5b target sequence is highlighted in yellow. The single nt mismatch in many of the DENV-1 isolates examined is highlighted in green. Shown on top is the sequence of the sh-5b target site, with the 5′ UAR sequence within it shown by the underline. (TIF) [file pntd.0001735.s002.tif]

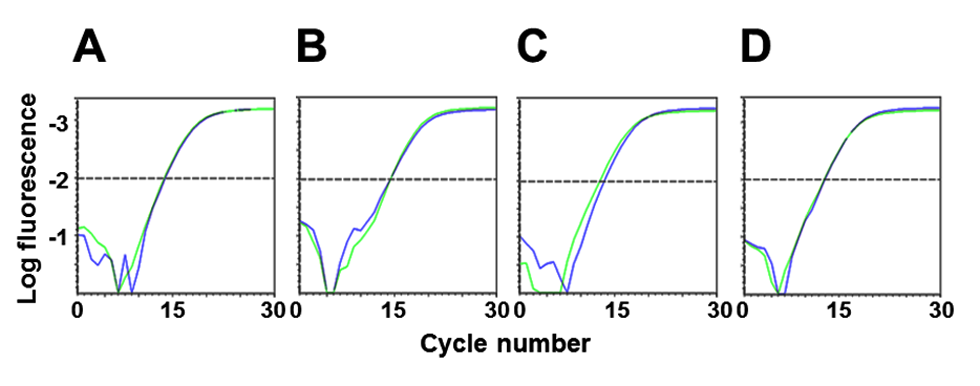

Supplement: Figure S3 — Real time PCR analysis of DENV RNA levels in Vero cells pre-infected with rAdsh-scr. Vero cells were either mock-pre-infected (blue curves) or pre-infected with rAdsh-scr (green curves) for 24 hours followed by infection with DENV-1 (A), DENV-2 (B), DENV-3 (C) and DENV-4 (D). Total cellular RNA was isolated on day 7 post-DENV infection and analyzed for DENV ‘plus’ sense genomic RNA by real time PCR. The horizontal dashed line indicates the baseline used to determine Ct values. (TIF) [file pntd.0001735.s003.tif]
